# Supplementary material for: Community perceptions of citizen science approach in pandemic preparedness and response in South and Southeast Asian countries
Source: PLOS Glob Public Health. 2025 Aug 21;5(8):e0003696. doi: 10.1371/journal.pgph.0003696 (PMC12370037; doi:10.1371/journal.pgph.0003696)
Supplement: S1.Text — (PDF) [file pgph.0003696.s001.pdf]

Thank you everyone for taking time to participate in our focus group discussion. All of you have participated in our survey previously looking at citizen science. For our discussion today, we would like to discuss in more details some of the findings from the survey. We would also like to hear your thoughts, preferences, and motivators to participate in citizen science activities as well as discuss more about the idea of digital citizen science.

What you say during this discussion will help us in the conceptualization and design of citizen science activities in the future. If there are questions that you do not feel comfortable talking about, please skip them. As this is a group discussion, please feel free to pick up on each other's points but also be respectful when others are talking. There are no right or wrong answers and we are all here to learn from each other.

Before we start, we would like everyone here to keep the details of this discussion confidential. What you say during this discussion will be audio recorded for analysis purposes, but please be assured that all data will be kept confidential, and all names mentioned will be removed during the analysis. Do you have any questions before we begin?

1. Let us start the discussion by first talking about the concept of citizen science. From the survey, we found that you relate these words to the term citizen science. [*print out word cloud of CS phrases and show to participants*] Can you explain how you relate to these words and why you think they describe citizen science?
    a. Imagine that now you have to explain citizen science to a fellow friend/peer, what would you say in your own words and how would you do it? [probe for slogan, medium to use]
2. In the survey, we also explored some of the citizen science activities related to managing pandemics in *your country [replace with country name]*. Some examples mentioned included [*insert activities based on slide 5*]. For these activities, can you share more about how you have been involved, elaborating on your role and the processes and tasks involved? [*for those who have not been involved, facilitator could ask them to imagine how they would like to be involved and what is the ideal state*]
    a. What did you like about the experience?
    b. What did you not like about the experience? How can it be improved?

    c. Are there other activities which you can think of that have not been mentioned? Please elaborate.

3. According to the survey, most people agreed that it is important/useful to involve the community in science/research activities for managing pandemics/outbreaks.
    a. What role(s) do you think citizens/the public should play?
    b. What advantages can you think of when involving the community in science/research activities for managing pandemics/outbreaks?
    c. What disadvantages can you think of when involving the community in science/research activities for managing pandemics/outbreaks?
4. What are the top 3 reasons that will motivate you to participate in pandemic/outbreak-related citizen science activities?
    a. **Why** these reasons?
5. What are the top 3 reasons that will discourage/stop you from participating in pandemic/outbreak-related citizen science activities? [*possible probes: work/study pressure, family pressure, health issues, communication*]
    a. **Why** these reasons?
6. Of the following categories of reasons that will determine your participation in pandemic/outbreak-related citizen science activities [*print out the table of categories and show to participants*], please rank them according to which you think is the most important reason to the least important reason. Please explain **why**.
7. One of the main objectives of citizen science is to hear the voices of the community and allow community members, researchers, and policymakers to define problems together and work on solutions together. What do you think of such an approach?
    a. In terms of having your voices heard, how do you envision this being done? [*probe for the entire process from defining problem to co-designing the solution*]
        i. What will be your main wishes and concerns?
    b. Are there any other concerns when interacting with researchers and policymakers?
8. According to the survey, most people will be interested to participate in citizen science activities through digital platforms instead of traditional paper form or in-person interactions. Please tell us more about **why** this is so.
    a. What are some of the advantages of each method of engagement?
    b. What are some of the disadvantages of each method of engagement?

9. What types of pandemic/outbreak-related citizen science activities do you think can be done digitally/online?
10. According to the survey, most people expect to gain/learn something out of their participation in citizen science activities. Can you help us understand what do you hope to gain/learn?
    a. **Why** is this important to you?
11. Do you think participating in citizen science activities is sustainable in the long run? Please **explain**.
    a. If no, what will encourage you to continue your participation in such activities and why so?
    b. If yes, why so?
12. What resources do you think are needed for you to participate in pandemic/outbreak-related citizen science activities in the long run?

We have come to the end of our discussion. Are there any questions that you would like us to clarify? I would like to thank you all for your participation.
