# Supplementary material for: Community perceptions of citizen science approach in pandemic preparedness and response in South and Southeast Asian countries
Source: PLOS Glob Public Health. 2025 Aug 21;5(8):e0003696. doi: 10.1371/journal.pgph.0003696 (PMC12370037; doi:10.1371/journal.pgph.0003696)
Supplement: S1 Fig — (PDF) [file pgph.0003696.s002.pdf]

CITIZEN SCIENCE

refers to the practice of active public participation, collaboration, and co-creation in all aspects of scientific research.

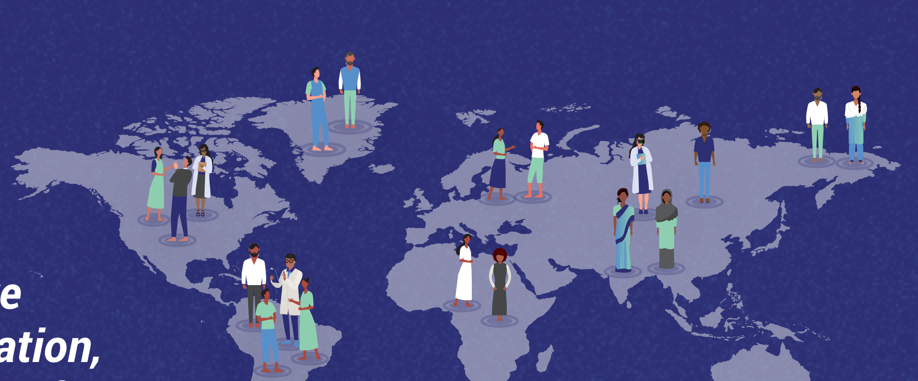

[redacted]

Citizens, like you, can work with scientists, participate in research and even assist authorities in mitigating health crises, without any particular scientific background.

LEVELS OF ENGAGEMENT

Depending on your level of engagement, there are different research activities you can take part in, as citizen scientist.

LEVEL 1:

You are **consulted** by professional scientists through research questions, neighborhood meetings or public hearings.

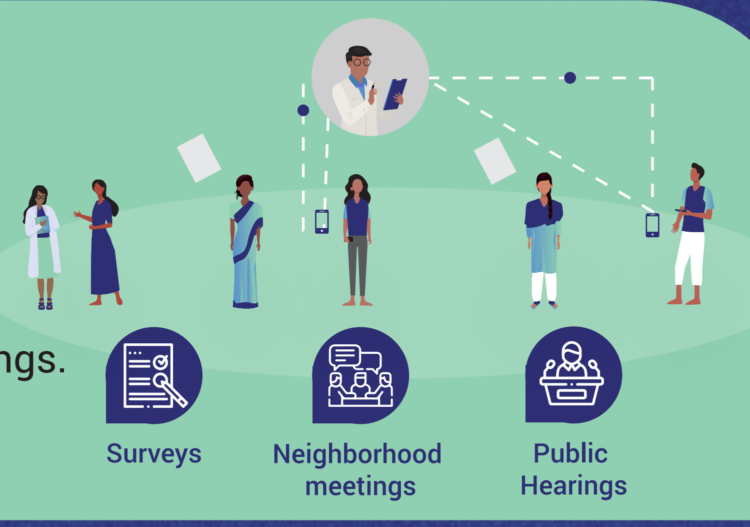

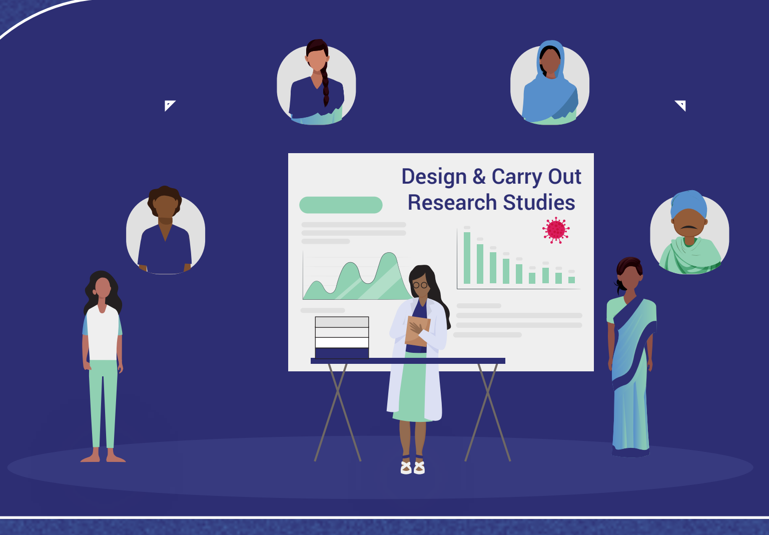

LEVEL 2:

You **participate** in the design and execution of research studies.

LEVEL 3:

You **work** more closely with the researchers and become contributors of knowledge, expertise and experience.

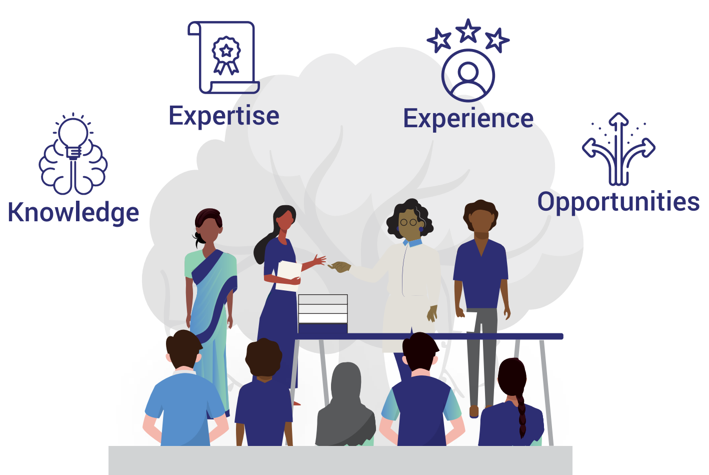

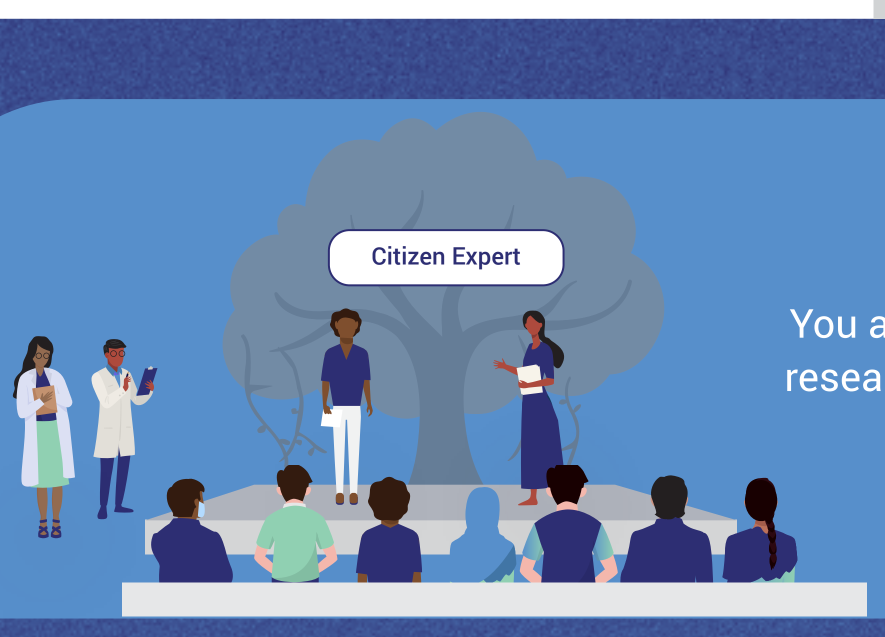

LEVEL 4:

You are fully empowered to research problems and implement solutions according to your community's needs.

STAGES OF A PANDEMIC

As citizen scientist, You can work with professional scientists and authorities to prepare and respond better against new and emerging infectious disease outbreaks.

When a pandemic is declared, mobilizing the public to swiftly and effectively respond to its various stages can save lives.

STAGE 1:
PRE-PANDEMIC PHASE

As citizen scientist, you can facilitate community engagement in the event of a local outbreak, by building trust and growing collective habits in your community. It will reduce the risk of the local episode becoming global.

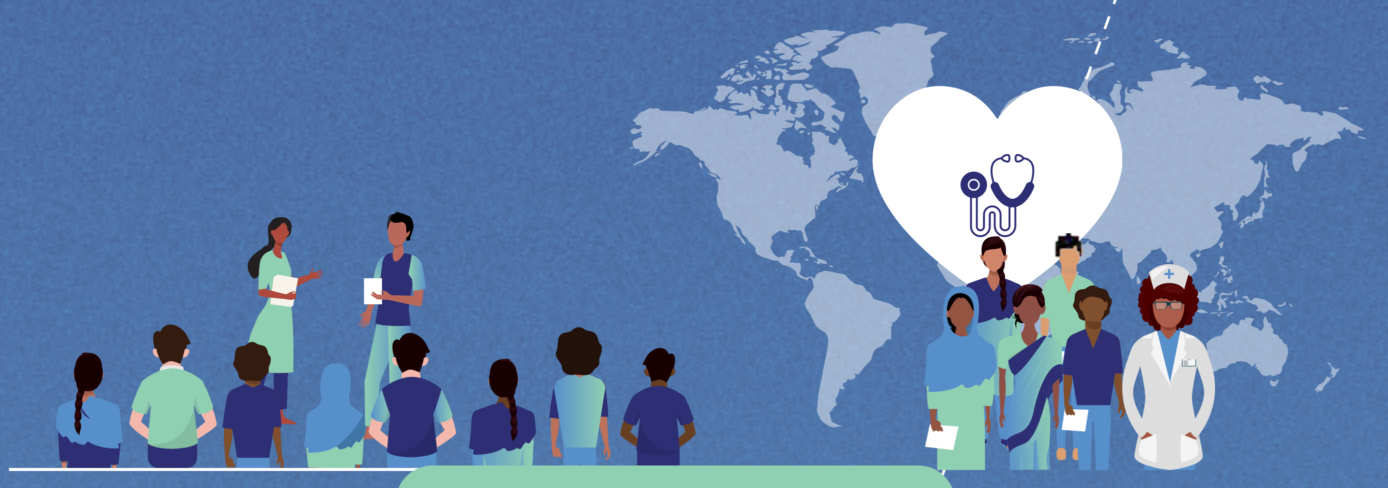

STAGE 2:
THE ALERT PHASE

You can be involved at the centre of a surveillance system to identify and monitor diverse data sources across the human-animal-environment interface ('One Health approach').

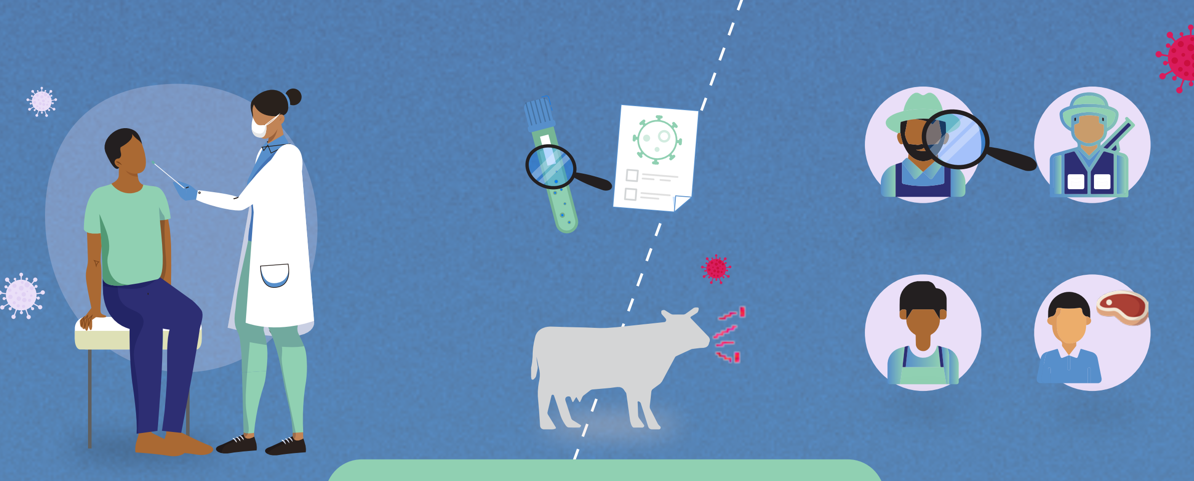

STAGE 3:
THE PANDEMIC PHASE

You can participate in generating models of outbreak outcomes, and tailored visualizations and communications about the pandemic, by contributing data and vision grounded in your reality. This knowledge will inform crucial decisions in the management of the pandemic.

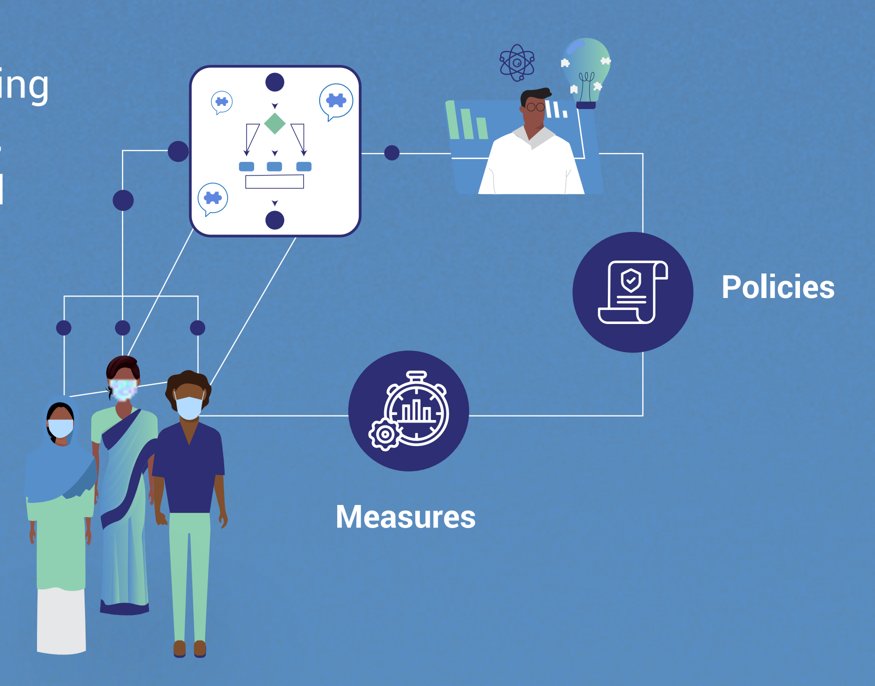

STAGE 4:
THE TRANSITION PHASE

Your observations on the long-term health effects of the infection and potential side effects of the deployed cure will help researchers develop better solutions.

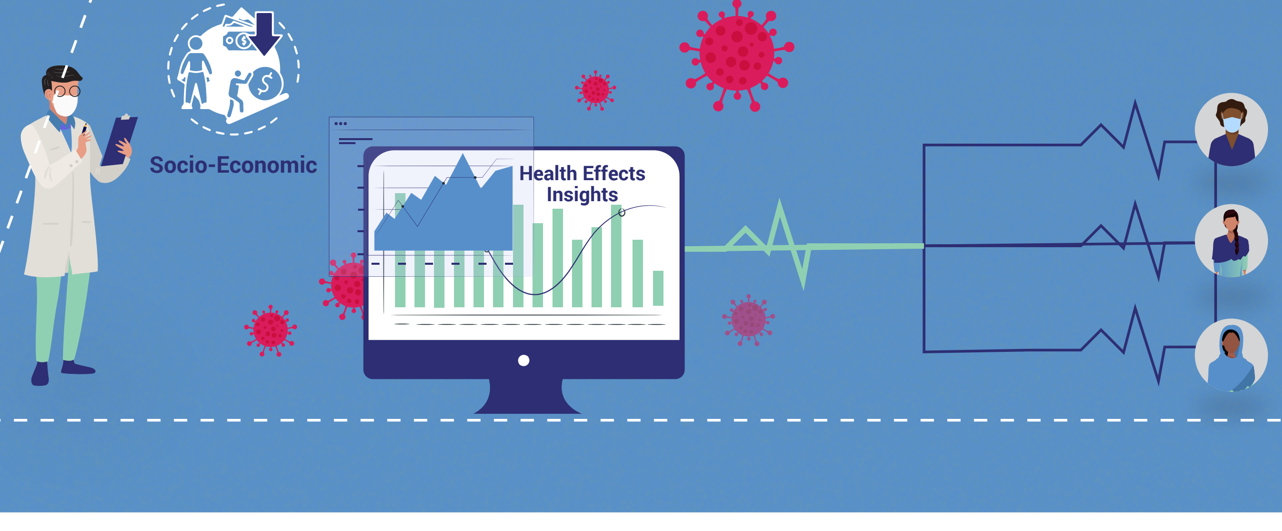

PARTICIPATORY APPROACHES

I-DAIR recognizes community members as not only data providers, but important knowledge experts and by creating an equal partnership between communities and scientific institutions, we can collectively prepare and respond better to the next pandemic.

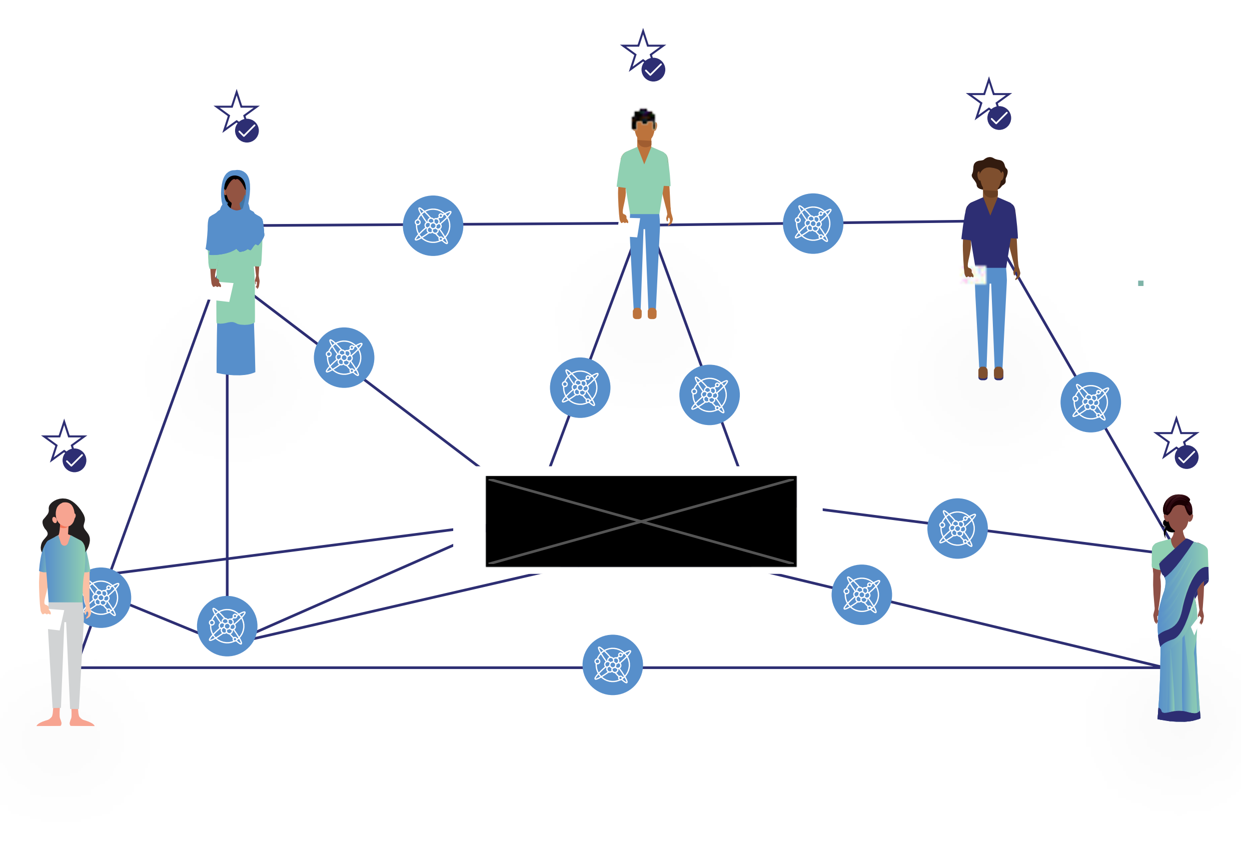

[redacted]
