# Supplementary material for: Community perceptions of citizen science approach in pandemic preparedness and response in South and Southeast Asian countries
Source: PLOS Glob Public Health. 2025 Aug 21;5(8):e0003696. doi: 10.1371/journal.pgph.0003696 (PMC12370037; doi:10.1371/journal.pgph.0003696)
Supplement: Attachment — Submitted filename: Comments and Response_28_2024.doc [file pgph.0003696.s004.doc]

PGPH-D-24-01917: Qualitative assessment of the citizen science approach to foster public partnerships for pandemic preparedness and response in South and Southeast Asian countries.

**Reply to the Editor/Reviewers’ comments**

We sincerely thank the editor and reviewers for their valuable repeated efforts for providing their valid and constructive comments in order to improve manuscript. We agreed with all suggestions and accordingly changes have been made. They are reflected with text highlighted with track changes in MARKED DOCUMENT.

Responses specific to comments are mentioned below for check;

| Original comments of the Editor/reviewer | Reply by the author(s) | Changes done on page number and line number  (PLEASE CHECK MARKED DOCUMENT) |
| --- | --- | --- |
| **Editor** |  |  |
| Please include a complete copy of PLOS’ questionnaire on inclusivity in global research in your revised manuscript. Our policy for research in this area aims to improve transparency in the reporting of research performed outside of researchers’ own country or community. The policy applies to researchers who have travelled to a different country to conduct research, research with Indigenous populations or their lands, and research on cultural artefacts. The questionnaire can also be requested at the journal’s discretion for any other submissions, even if these conditions are not met.  Please find more information on the policy and a link to download a blank copy of the questionnaire here: <https://journals.plos.org/globalpublichealth/s/best-practices-in-research-reporting>. Please upload a completed version of your questionnaire as Supporting Information when you resubmit your manuscript. | Included | Separate file (Others Category) |
| Please amend your detailed Financial Disclosure statement. This is published with the article. It must therefore be completed in full sentences and contain the exact wording you wish to be published.  1. Please clarify all sources of funding (financial or material support) for your study. List the grants (with grant number) or organizations (with url) that supported your study, including funding received from your institution.  2. State the initials, alongside each funding source, of each author to receive each grant.  3. State what role the funders took in the study. If the funders had no role in your study, please state: “The funders had no role in study design, data collection and analysis, decision to publish, or preparation of the manuscript.”  4. If any authors received a salary from any of your funders, please state which authors and which funders. | Mentioned | Page-21 Lines: 479-83 |
| In the online submission form, you indicated that "Data can be provided upon request.". | It has now changed in submission form,  Please check. |  |
| We have noticed that you have uploaded Supporting Information files, but you have not included a list of legends. Please add a full list of legends for your Supporting Information files after the references list. | Included | Page-28; Lines648-52 |
| Supplementary 2_Citizen science infographics.pdf: Please confirm whether you drew the images / clip-art within the figure panels by hand. If you did not draw the images, please provide (a) a link to the source of the images or icons and their license / terms of use; or (b) written permission from the copyright holder to publish the images or icons under our CC-BY 4.0 license. Alternatively, you may replace the images with open source alternatives. See these open source resources you may use to replace images / clip-art:  - [https://commons.wikimedia.org](https://commons.wikimedia.org/)  - <https://openclipart.org/> | The images in the infographics are produced by a Creative and Digital Experience Agency called Motionify, which I-DAIR has engaged to help produce the infographics. The infographics and the citizen science video are all open access and can be found on I-DAIR’s website (https://www.healthai.agency/blog/citizen-science-for-pandemic-response) | Supplement-2  Page: 28; Line: 650-2 |
| Supplementary 1_FGD guide.docx and Supplementary 2_Citizen science infographics.pdf contains branding/a logo. We are not permitted to publish this under our CC-BY 4.0 license, even with permission. We ask that you please remove or replace it. | Removed. Revised documents attached. | Supplement-1 |
| **Reviewer-1** |  |  |
| The manuscript has presented important problem. However, the scope hungs between the community involvement and Research dessimination. The abstract is not understandable and lacks consistency. The background is not focused and lacks strength. The methods section is incomplete for the types of the interview, how the interview was conducted who and how many of the data collectors had participated in the study, the data collection duration, place where the interview was conducted, the numbers of the interview per the interview types. In addition,the soft ware used for the analysis is missed. Above all, trustworthiness of the data is not ensured. The result is not systematically presented.The discussion fails to entail all its contents and strength. | Thank you for highlighting the importance of topic.  Abstract is restructured for clarity and consistency.  Background section is rewritten to make it focused.  Methods section:  The type of interview is focus group discussions, the number of interviews is mentioned in line…  Data collection duration is added.  It now includes interview settings and interviewer characteristics.  Data was analyzed manually by the study teams and no software was used for analysis.  Respective editing was done in results and discussion sections | Page-2-3 (Lines: 31-70)  Page-4-7 (Lines: 75-162)  Page-8 (Lines173-177)  Page-9 (Lines: 189-197)  Page-10 (Lines: 221-24)  Page: 17 (Lines: 371-80) |
| **Reviewer-2** |  |  |
| Introduction Start by framing the inadequacies of traditional approaches to pandemic management, particularly in terms of community trust and engagement. Then, CS will be introduced as a possible solution to these challenges. | Entire background section is rewritten as per the suggestions. Please check! | Page-4-7 (Lines: 75-162) |
| Repetitive Sentences and Ideas: Some ideas are repeated, particularly regarding the importance of community engagement and the potential of CS. Example: The sentence, “CS has piqued the interest of policymakers, program managers, and researchers...,” reiterates points already made earlier about the benefits of CS. Instead, consider combining similar points to streamline the argument. | The entire section has been rewritten and removed confusing and repetitive sentences. | Page-4-7 (Lines: 75-162) |
| Provide specific examples of how CS has been used successfully in similar contexts or how it could work in South and Southeast Asia to tackle pandemic-related challenges. | Mentioned in background section at different places to make continuity in section write-up. | Page-5: (Lines: 109-11)  Page-6 (Lines: 126-30)  Page-7 (Lines: 150-55) |
| While you mention the need to identify barriers to participation in CS, this section could be expanded. For example, what specific cultural, social, or economic barriers might exist in South and Southeast Asian countries that could impede the successful implementation of CS? Highlight any gaps in existing research that this study aims to fill. This will underscore the novelty and significance of the research. | The section has been rewritten based on comments | -do- |
| Method section  While the methodology is well-explained, it would benefit from additional justification for key decisions. For example, why were 4 focus groups chosen per country? Why was the decision made to exclude participants under 18 years or those with severe mental health conditions? | In each country, four focus group discussions (FGDs) were conducted with six to eight participants per FGD with thematic saturation achieved at the end of data collection. Thematic saturation was reached when no new themes emerged from the interviews.  Criteria to exclude individual to <18 years as they were adolescents whereas study focused on youths, whereas individuals with mental issues were excluded due to their inability to express their opinions during FGDs. | Page: 8 (Lines: 173-6)  Page: 8 (Lines: 184-6) |
| Some details about the data collection process could be expanded. For instance, how were the focus group discussions conducted? Were they virtual or in person? What measures were taken to ensure participants felt comfortable sharing their thoughts? Additionally, details on how the semi-structured guide based on the Precaution Adoption Process Model and Theory of Planned Behavior shaped the discussion would help the reader understand how these theories guided the research. | Now it has been clarified in detail for better clarity and understanding. | Page-9: (Lines: 190-9 & Lines: 202-8) |
| The section mentions "thematic saturation" but does not specify how saturation was assessed. This is crucial for qualitative research, as it justifies the number of FGDs conducted. | Added information on how thematic saturation was assessed, by looking at whether new themes emerged from the interviews. | Page-8 (Lines173-6) |
| **Reviewer-3** |  |  |
| There are some major discrepancies between the larger paper based on this mix-method research carried out by the same authors and published elsewhere (cited in reference 10) that includes five additional countries from Africa and this paper that includes only 5 countries from Asia and claims to be a qualitative study based on the 20 FGDs involving 130 individuals.  Some of these include the following: | It is right that mixed-methods study was done in more countries (Africa region) but current analysis was limited to south Asian countries as these regions differ in their socio-cultural context. This has been mentioned in study background with focusing objectives to South and South Asian Countries | Page: 17 (Lines: 376-80) |
| Definition of CS - the authors now seem to specifically include an additional dimension i.e. "to address public health challenges". They do not describe why the definition for the same study has changed between their publication in BMJ and the current submission. | It is rightly said, it was added as CS intends to provide understanding and solutions to public health challenges. However, it has been rectified to the same definition as the BMJ paper for concordance. | Page: 5 (Lines: 112-5) |
| Objectives of the study - This too has changed. how can the same study have different objectives between two separate publications; if the authors on the other hand note that the objectives of the present analysis of the larger study reported already through the BMJ report, then one can understand. But you cannot suddenly have new objectives with wider scope in the same study. | We have kept the same objectives; where it was focused on level of awareness, relatability, and acceptability; and level of readiness; and factors (facilitating and barriers) influencing participation. The same were now rewritten for concordance across manuscripts. | Page: 7 (Lines: 157-62) |
| Advantages and Disadvantages - Table 4 of the earlier publication (in BMJ) and Table 2 of the present submission in this respect seem to be coming up with different conclusions. To selectively draw only some responses out of 130 individuals and include in a table, appears to be somewhat far-fetched. Further, whereas the earlier publication classifies advantages and disadvantages by digital and analogue communications (and still reports verbatim comments of some respondents), the current report, although meant to be "Qualitative" according to the authors seems to be narrower than the earlier report - without any explanation. | Table 4 of earlier publication focused on comparison between analogue and digital methods of CS, which was already analyzed and reported in details, therefore we did not repeat this comparison analysis for this paper. Instead, Table 2 of current paper focused on perceived issues (advantages, disadvantages, facilitators, and barriers) of CS. This is corresponding to Table 3 in the BMJ paper where we looked at the quantitative analysis of these factors but did not analyze in details the qualitative component. Thus, the current paper attempted to discuss in more details the themes in this area and incorporated analysis of verbatims related to perceived issues of CS. All were analyzed as accordance to objectives and important verbatims were reported with respect to themes. | Page: 17 (Lines: 373-82) |
| Discussion / Conclusions - there seem to be some discrepancies between the discussion and conclusions of the earlier report and the present submission. The authors do not sufficiently explain these differences. Just to cite an example, the earlier report notes: "In Bangladesh, Indonesia, the Philippines, Cameroon and Kenya, majority were unaware of outbreak-related CS"; In the current report - the authors note "Across all countries, while referring to CS, the participants collectively comprehended the term "research". Participants understood that CS is possibly related to capacity building to learn and empower themselves in the field of research"; further they have also noted " It was also viewed as a social responsibility, especially in India. Distinctively, it was referred to as an awareness mechanism to improve knowledge about pandemic response in Bangladesh". There are similar distinctive differences and perceptions of contradictions between the earlier published report and the current manuscript. In my view, the authors have not done a thorough analysis of what they reported in the earlier publication based on the same work and what now they want to publish as a subset and based on FGDs only. At the minimum, they should attempt to quote what they wrote earlier and how and why it is different now. | There is difference in scope and depth of analysis in the two papers. As reported in BMJ, it was found that people were unaware about “outbreak-related CS” which is consistent with our reporting in the current paper. In the current paper, although participants related CS to “research” as a term, they are still mostly unaware CS. What was described in the findings was their pereceived concept of CS. We have revised the findings section to correctly portray this. Current analysis states that some participants do participate/participated in various activities during COVID-19 but whether it was CS or not they do not know. What is evident in the current paper is that people are ready to participate further but certain support is warranted.  To reiterate, the current paper did a more in depth analysis of the qualitative data to have a deeper understanding of the lived experiences of citizens and their potential roles in pandemic preparedness and response, as well as their perspectives towards CS and the factors which will affect their participation in CS activities. We have highlighted this in the discussion to differentiate between the two papers and emphasize the value add of the current paper.  We argue that the current paper presented more detailed analyses which was limited in BMJ manuscript where it was both quantitative and qualitative (the qualitative segment was limited to definition of CS and comparing methods of CS delivery). It warranted a separate qualitative analysis in order to better understand the narratives about and around CS. | Page-17 (Lines: 372-82) |
