# Supplementary material for: Community perceptions of citizen science approach in pandemic preparedness and response in South and Southeast Asian countries
Source: PLOS Glob Public Health. 2025 Aug 21;5(8):e0003696. doi: 10.1371/journal.pgph.0003696 (PMC12370037; doi:10.1371/journal.pgph.0003696)
Supplement: Attachment — Submitted filename: Comments and Response_01_2025.doc [file pgph.0003696.s006.doc]

PGPH-D-24-01917: Qualitative assessment of the citizen science approach to foster public partnerships for pandemic preparedness and response in South and Southeast Asian countries.

**Reply to the Editor and Reviewers’ comments**

We sincerely thank the editor and reviewers for their valuable repeated efforts for providing their valid and constructive comments in order to improve manuscript. We agreed with all suggestions and accordingly changes have been made. They are reflected with text highlighted with track changes in MARKED DOCUMENT.

Responses specific to comments are mentioned below for check;

| Original comments of the Editor/reviewer | Reply by the author(s) | Changes done on page number and line number  (IN MARKED DOCUMENT) |
| --- | --- | --- |
| **Editor** |  |  |
| Reviewer 3 noted discrepancies between Table 4 in your BMJ publication and Table 2 in this manuscript, including differences in conclusions and selective use of responses. Please explain the methodology used for Table 2, clarify why the scope appears narrower, and justify more in details how this analysis builds on or diverges from the earlier report. | We have revised it accordingly. To further clarify, Table 4 in our BMJ publication focused on the comparison between digital and analogue CS, whereas Table 2 in this paper focused on the lived experiences of participants when exploring the factors influencing participation and sustainability of CS. In our BMJ paper, the quantitative component was mainly used to triangulate the quantitative findings, without going into in-depth analysis. This paper aimed to expand on that by exploring in more details the factors mentioned by participants particularly in the South and South Asian Countries. | Page-14-15  Lines: 307-24 |
| Discussion/Conclusions discrepancies: Reviewer 3 highlighted differences in how CS was perceived across countries in your BMJ paper and the current submission. Please quote the earlier findings and provide a thorough explanation for these differences with the current findings, emphasizing how this work adds value. | We have revised it accordingly | Page-14-15  Lines: 307-24 |
| From the comments above, Reviewer 3 remains unconvinced that the revision in Lines 372–383 sufficiently shows how this manuscript provides additional detail beyond the BMJ paper. Please expand this discussion with specific and clear examples to strengthen your argument. | We have revised it accordingly | Page-14-15  Lines: 307-24 |
| Please correct typographical errors (e.g., "county" instead of "country" on line 240) and ensure the rest of the manuscript is error-free. | Express apology for typing error. It is corrected | Page-7,  Line 173 |
| **Reviewer-3** |  |  |
| While authors have tried to explain their logic, i am not fully convinced with their answers or their response that this is more detailed analysis compared to the BMJ paper. Lines 372 to 383, according to them responds to some of the concerns expressed earlier. But I am not fully convinced. | It is to clarify that in the BMJ paper, comparison was done between analogue and digital methods of CS. That paper focused on the potential applicability of digital methods in implementing CS activities. Whereas, current paper focused on perceived factors (facilitating/inhibiting) affecting CS implementation, concerns of people while interacting with stakeholders (researchers and policy makers), and factors related to sustainability of CS. Therefore, current paper did not limit to comparison of digital with analogue method but shared citizens’ lived experiences while describing CS as a concept and its potential in pandemic preparedness and control. | Page-14-15  Lines: 307-24 |
| New mistakes (line 240 - county instead of country) have also crept in. | Express apology for typing error. It is corrected | Page-7,  Line 173 |
| I am as such unable to make a new recommendation as I believe they do not respond to the concerns expressed adequately | We have revised the particular and important concern. You are requested to consider this. | Page-14-15  Lines: 307-24 |
