# Supplementary material for: Community perceptions of citizen science approach in pandemic preparedness and response in South and Southeast Asian countries
Source: PLOS Glob Public Health. 2025 Aug 21;5(8):e0003696. doi: 10.1371/journal.pgph.0003696 (PMC12370037; doi:10.1371/journal.pgph.0003696)
Supplement: Attachment — Submitted filename: Reviews for the manuscript - PLOS.docx [file pgph.0003696.s005.docx]

Reviews for the manuscript “PGPH-D-24-01917R2”

1. **Summary of the research**

This paper details the findings of focus group discussions in five countries with citizens of each to assess their perceptions of the usefulness, benefits and challenges of using CS for pandemic preparedness and response. While not entirely novel, the findings are interesting and can add value to the current literature on CS and community health. I also enjoyed reading the respondents’ quotations, which provide valuable insights into the topic.

The main argument of this study is that CS might be a viable approach for potential disease prevention efforts by learning from respondents’ experience of the Covid-19 pandemic, and how they believe CS can contribute to future public health crises. This is mentioned in lines 315-318 but is not clearly signposted from the beginning and throughout the paper. The abstract, for instance, makes no specific mention of this.

2. Examples and evidence

**Major issues**

1. Lack of context on citizen science

This study falls short of providing adequate context for the application of citizen science in pandemic preparedness and response, and in terms of addressing societal issues at the community level. For instance, citizen science has been used with some success to address community health issues in relation to water and air pollution, in environmental monitoring, in food security issues and in disease prevention. Reference to these studies could add context and underscore the significance and benefit of using citizen science for pandemic preparedness.

In the tables, participants refer to CS as being able to overcome geographic and other limitations to data access (Table 1, Participation in Research, Data generation and analysis) and enabling bottom-up research (Table 2, People-centric, Realistic approach). These are among the benefits of CS, which are not adequately discussed in the paper.

2. Lack of justification for the selection of countries

Five countries are included in this paper. However, no explanation is given for why these five countries were chosen. There is a mention of this study being part of a larger study, findings of which has been published in a separate paper (see line 109). In that paper, it was mentioned that these countries were chosen at least partly to fill the gap in the literature, which has so far focused on high income countries. It would be beneficial to include the same information in this paper.

3. Lean into the demographic of the participants

There is an opportunity here to highlight that the demographic of the participants in this study (the youths and marginalized and indigenous populations) fills the gap in many CS studies, which have shown that these groups are generally excluded from CS as citizen participants as well as the subjects of CS projects. Please refer to published papers that have examined the demographics of CS volunteers.

**Minor issues**

1. Include quotations in the findings and discussions rather than highlighting them only in the tables, to make the texts more engaging and interesting.
2. There are some sentences that are truncated and incomplete. See e.g lines 87-90, 91-92 and 94-97.
3. Unclear focus on youth

In line 114-115 it is mentioned that the study involved youths (participants were purposively sampled to include youths…”) as well as other groups. However in line 121-123, youths were highlighted as if they were the sole focus of the study (“As study focused on youths…”). This inconsistency can be confusing and misleading.

4. General population

Unclear what the general population who make up the study sample refer to. Please clarify.

5. The study refers to the infographic and videos used to explain to participants what CS is. These are linked in the document to external resources. For the benefit of text readers, it would be useful to include a brief description of the content of these materials within the text.
6. Title of the paper may perhaps be clearer about the focus of the study. “Qualitative assessment of the CS approach…” is quite vague. “Community perceptions of the CS approach in pandemic preparedness” provides the reader with immediate and clear information about the study.

**Other points (for editors only)**

There are references in this paper to the authors’ previous publication in the BMJ, which is also mentioned by the previous reviewers. I managed to get access to the BMJ paper to get additional context for this study. Not all readers of this publication might have access to that article, however, and is quite confusing and time-consuming to jump back and forth. Perhaps the authors can add some highlights of the BMJ paper, so this paper can be read as a stand-alone.
