# Supplementary material for: Community perceptions of citizen science approach in pandemic preparedness and response in South and Southeast Asian countries
Source: PLOS Glob Public Health. 2025 Aug 21;5(8):e0003696. doi: 10.1371/journal.pgph.0003696 (PMC12370037; doi:10.1371/journal.pgph.0003696)
Supplement: Attachment — Submitted filename: Comments and Response_03_2025.doc [file pgph.0003696.s007.doc]

PGPH-D-24-01917: Community perceptions of citizen science approach in pandemic preparedness and response in South and Southeast Asian countries (Revised based on reviewer comment)

**Reply to the Editor and Reviewers’ comments**

We sincerely thank the editor and reviewers for their valuable repeated efforts for providing their valid and constructive comments in order to improve manuscript. We agreed with all suggestions and accordingly changes have been made. They are reflected with text highlighted with track changes in MARKED DOCUMENT.

Responses specific to comments are mentioned below for check;

| Original comments of the Editor/reviewer | Reply by the author(s) | Changes done on page number and line number  (IN MARKED DOCUMENT) |
| --- | --- | --- |
| **Reviewer-5** |  |  |
| This paper details the findings of focus group discussions in five countries with citizens of each to assess their perceptions of the usefulness, benefits and challenges of using CS for pandemic preparedness and response. While not entirely novel, the findings are interesting and can add value to the current literature on CS and community health. I also enjoyed reading the respondents’ quotations, which provide valuable insights into the topic. | We appreciate the encouraging observation made by the reviewer along with valuable comments to further improve the manuscript to improve its value in existing literature. We are happy that reviewer enjoyed while going through quotations which are reflective of manuscript. | Not Applicable |
| The main argument of this study is that CS might be a viable approach for potential disease prevention efforts by learning from respondents’ experience of the Covid-19 pandemic, and how they believe CS can contribute to future public health crises. This is mentioned in lines 315-318 but is not clearly signposted from the beginning and throughout the paper. The abstract, for instance, makes no specific mention of this. | You have correctly summarized the entire manuscript and we completely agree with your observations. We have made all necessary changes in line to main argument, as suggested.  You are requested to please go through the itemized responses below as well. | Entire manuscript including abstract. |
| a) Lack of context on citizen science  This study falls short of providing adequate context for the application of citizen science in pandemic preparedness and response, and in terms of addressing societal issues at the community level. For instance, citizen science has been used with some success to address community health issues in relation to water and air pollution, in environmental monitoring, in food security issues and in disease prevention. Reference to these studies could add context and underscore the significance and benefit of using citizen science for pandemic preparedness. | We agree and have studied the applications of CS in health and non-health sectors and included in background of study. | Introduction Section:  Page: 4  Lines: 80-87 |
| In the tables, participants refer to CS as being able to overcome geographic and other limitations to data access (Table 1, Participation in Research, Data generation and analysis) and enabling bottom-up research (Table 2, People-centric, Realistic approach). These are among the benefits of CS, which are not adequately discussed in the paper. | We have revised the discussion where all findings are discussed including Table-1 and 2, as mentioned | Discussion Section:  Page: 16-18 |
| b) Lack of justification for the selection of countries  Five countries are included in this paper. However, no explanation is given for why these five countries were chosen. There is a mention of this study being part of a larger study, findings of which has been published in a separate paper (see line 109). In that paper, it was mentioned that these countries were chosen at least partly to fill the gap in the literature, which has so far focused on high income countries. It would be beneficial to include the same information in this paper. | It is correctly stated that the selection of countries was to fill the gap by providing evidence from low-and middle-income countries (LMIC). So, we have included the justification of selection of country in revised manuscript. | Methods section  Page-8, Lines: 134-6. |
| **c**) Lean into the demographic of the participants  There is an opportunity here to highlight that the demographic of the participants in this study (the youths and marginalized and indigenous populations) fills the gap in many CS studies, which have shown that these groups are generally excluded from CS as citizen participants as well as the subjects of CS projects. Please refer to published papers that have examined the demographics of CS volunteers. | It is correct that it needs to be mentioned in discussion section where said segment of population are excluded in studies. The same has been incorporated in the discussion section. | Discussion section:  Page-17  Lines: 333-40 |
| **Minor Issues** |  |  |
| a) Include quotations in the findings and discussions rather than highlighting them only in the tables, to make the texts more engaging and interesting. | It is great idea but doing this is going to increase the word limit. As all quotes are arranged to themes and subthemes so mentioning even few quotes in findings and discussion will give suboptimal picture of data/information.  We apologize for the same! | --- |
| b) There are some sentences that are truncated and incomplete. See e.g lines 87-90, 91-92 and 94-97. | We have now revised the entire background section focusing the main argument (please refer second comment) as suggested by reviewer. | Background section:  Page-4-6 |
| c) Unclear focus on youth In line 114-115 it is mentioned that the study involved youths (participants were purposively sampled to include youths…”) as well as other groups. However in line 121-123, youths were highlighted as if they were the sole focus of the study (“As study focused on youths…”). This inconsistency can be confusing and misleading. | It was an error and has been removed. | Methods Section:  Page:8  Line: 150 |
| d) General population Unclear what the general population who make up the study sample refer to. Please clarify. | Clarified | Methods section:  Page:7  Line: 139 |
| e) The study refers to the infographic and videos used to explain to participants what CS is. These are linked in the document to external resources. For the benefit of text readers, it would be useful to include a brief description of the content of these materials within the text. | We have mentioned | Methods Section:  Page: 10  Lines181-3 |
| f) Title of the paper could perhaps be clearer about the focus of the study. “Qualitative assessment of the CS approach…” is quite vague. “Community perceptions of the CS approach in pandemic preparedness” provides the reader with immediate and clear information about the study. | We have changed it accordingly | Page-1  Lines: 1-3 |
