# Supplementary material for: Community perceptions of citizen science approach in pandemic preparedness and response in South and Southeast Asian countries
Source: PLOS Glob Public Health. 2025 Aug 21;5(8):e0003696. doi: 10.1371/journal.pgph.0003696 (PMC12370037; doi:10.1371/journal.pgph.0003696)
Supplement: Attachment — Submitted filename: Comments and Response_05_2025.doc [file pgph.0003696.s008.doc]

PGPH-D-24-01917: Community perceptions of citizen science approach in pandemic preparedness and response in South and Southeast Asian countries (Revised based on reviewer comment)

**Reply to the reviewers’ comments**

Dear Editor,

We thank you for providing the opportunity to revise and submit the manuscript. We are thankful to the reviewers for their feedbacks and comments to improve the manuscript. We sincerely appreciate their valuable repeated efforts for providing their valid and constructive comments in order to improve manuscript. We agreed with suggestions accordingly and changes reflected with text highlighted in track changes.

Here is our pointwise response the reviewers’ comments and concerns.

| Original comments of the reviewers | Response by the authors |
| --- | --- |
| **Overall** |  |
| The authors are requested to prepare the response file by showing the revised changes directly in the response file, instead of only providing line numbers. This will facilitate the review of the changes made in the manuscript. In case of major changes, line numbers may be mentioned only. | Thank you for the suggestion, we have now included revised changes in the response file. |
| It would be helpful to avoid abbreviations throughout the document, as they interrupt the reading flow. | It is a very good advice; we have attempted to remove the unwanted abbreviations for smooth reading. |
| **Introduction** |  |
| Lines 85 to 87: Please expand the term 'diverse geographical settings.' It would be helpful to mention the names of the geographical settings referenced here. | It is mentioned in revised manuscript |
| Line 88 to 90: lease expand the sentence further. From the current description, it is unclear what significant variations you are referring to and how they are connected to your paper’ | The same has been clarified that how citizen science was viewed and what kind of variation exists in understanding, interpretation, and implementation |
| **Methods** |  |
| The Methods section should include separate paragraphs for each theme, for example, study site and design, participant selection, data collection, etc. This will improve the clarity of the Methods section, which is currently somewhat difficult to follow. It is also important to ensure transferability of this research. | Subsection of methods are now included in the revised manuscript as;  Study design and site, study participants, selection of participants, data collection, data analysis, and ethical approval |
| The participant selection criteria need to be clarified further. While the type of group is defined, it is not clear how and from where the participants were selected. Although the study country is specified, it is important to explain how the researchers chose the specific study location and how participant selection was carried out. In qualitative research, providing a clear description of participant selection is essential to ensure rigor and support transferability. | The subsection “selection of participants” is now included in revised manuscript explaining how they were recruited. |
| A survey is mentioned in the Methods section; providing a bit more detail about this survey would help readers better understand the context. It would also clarify the participant selection process. | The same has been now mentioned in methods subsection “selection of participants”. |
| It is stated that the staff who collected data had good rapport with the community, but it is not clear how this rapport was developed or what the process of becoming familiar with participants involved. Additionally, since participants were interviewed in a hall or health center, it should be clarified how a ‘natural setting’ was maintained during the interviews. | As mentioned, in each country, selected Community Based Organizations (CBOs) were already working for people along with health system, other self-help groups, and community representatives. Thus, they already had a good rapport with population in their working areas. The same has been mentioned in the methods section. |
| The information on data saturation should be included in the analysis section rather than in the description of the tools. | We agree, the same has been moved to “Data collection” subsection under methods. |
| The Methods section mentions the use of PAPM and TPB; however, a more detailed explanation linking these frameworks to the objective of the manuscript is important. For example, in lines 167 and 168, 'a series of stages' is mentioned, but it is unclear how this was connected to the FGD guides, the analysis, or how it is presented in the results. Elaborating on this would help demonstrate better relevance. | The PAPM and TPB model were used to devise questions where individual beliefs, attitude, concerns, and experiences were asked to elicit their intention and participating behavior. The same has been edited in “data collection” subsection in methods section. |
| Lines 170 to 184 mention 'awareness, relatability, acceptability, feasibility, and sustainability,' but the descriptions in the supplementary file differ. It would be helpful to provide a brief description of the themes and subthemes in the Methods section rather than referring only to the supplementary tables. | The PAPM and TPB model were used to devise questions, whereas themes and subthemes were results based on participant’s narrative. The suggested changes have been made in “data analysis” subsection in methods section. |
| Lines 178 to 179: The sentence 'Venues to conduct FGD…' should be moved to the section where venue-related information is provided (from line 160 onwards). | The same has been moved to “data collection” subsection in methods section. |
| Lines 180 to 184: It would be helpful to define or specify the 'various stages' of citizen engagement. | It is specified in “data collection” subsection in methods section. |
| The analysis procedures followed should be clearly described, with appropriate references, rather than simply mentioning 'content analysis’. | The same has been clarified along with reference (no. 20) |
| It is also important to specify the type of consent obtained—whether written or verbal. | It was a written informed consent and same is mentioned |
| **Results** |  |
| A table presenting the demographic profile of all participants is required; otherwise, it is difficult to connect the findings to the participants’ context. The demographic information may include age, education, occupation, or any other background details relevant to understanding the context of their responses. | We acknowledge the importance of demographics in providing context to the findings. However, as the study population was more sensitive, such as the marginalized group, the study team decided at the study design stage not to collect any demographics information. This was advised by the local study teams given their past experiences interacting with the local people. This helped to make the participants feel more at ease and willing to share. Instead, we included the study site, and population group in the findings to provide the relevant context to the findings.  The same has been included in “data collection” (Page: 9) subsection and discussed as a limitation in discussion section (Page: 20) |
| Lines 221 to 222: The sentence 'Domains of FGD are summarized by the theme…' should be moved to the analysis section of the Methods, as it reflects methodological details rather than results. | The same has been moved to “data analysis” subsection of methods. |
| Lines 232 to 235: The terminology 'awareness mechanism' is unclear; it would be helpful if the authors could revise or clarify its meaning. | It is now clarified accordingly in subsection “a” of results section (page-10) |
| In several places in the Results section, terms such as 'majority' and 'most' are used. It is important to define these terms—either in the Methods section or in the Results—by indicating the number of responses out of the total participants in parentheses. | The percentages of number of participants responded have been indicated in parenthesis. |
| **Discussion** |  |
| The first sentence of the Discussion should present the main findings connected to the main objective of the research (mentioned in last sentence of the introduction). | The main findings of the study are included as first sentence of discussion section. |
| Lines 333 to 338: This sentence will be better justified if the authors provide a clear description of the rationale for participant selection in the Methods section and connect the results more explicitly to these different categories. | The rationale is given in “Selection of participants” subsection of methods section. |
| Lines 340 to 350: The sentence 'it is mentioned that… in analogue form' seems more like a rationale and should be moved to the Introduction section. | The same has now moved to introduction section. |
| Lines 360 to 378: This description appears to be a repetition of the results. It would be helpful if the authors could link the findings with existing literature and discuss them further. | The repetition has been omitted and finding of current study is linked with existing literature. (Please see page 16-17) |
| Lines 439 to 446: To describe this adequately, a detailed and clear explanation of the participant selection methods is crucial. Without this, the rigor of the study will be difficult to assess. | The same is mentioned in “Selection of participants” subsection of methods section. |
| Lines 466 to 469: The recommendation regarding methodological strengths discussed throughout the Discussion section is missing. The authors may consider adding it alongside the current recommendations. | It is a very good suggestion. A line of recommendation is now included in conclusion section |
| **Tables** |  |
| The term 'Sir' is mentioned in several places, indicating a hierarchy between the participants and the researcher. This suggests that rapport building may not have been optimal, and the natural setting was interrupted, which could have led to biased responses. It would be helpful to clarify this in the Methods section and address it as a limitation. | It is to mention that the term “Sir” was used in Indian setting as a “greeting gesture” to one of investigator, as a culture. It is used by some, not by all participants. It has not limited the rapport as participants have spoken and shared their viewpoints. As you are right that it might look distinct as compared to rest of quotes, so same has now being removed. |
