# Supplementary material for: Community perceptions of citizen science approach in pandemic preparedness and response in South and Southeast Asian countries
Source: PLOS Glob Public Health. 2025 Aug 21;5(8):e0003696. doi: 10.1371/journal.pgph.0003696 (PMC12370037; doi:10.1371/journal.pgph.0003696)
Supplement: Attachment — Submitted filename: Comments and Response_07_2025_2.doc [file pgph.0003696.s011.doc]

PGPH-D-24-01917: Community perceptions of citizen science approach in pandemic preparedness and response in South and Southeast Asian countries (Revised based on reviewer comment)

**Reply to the reviewers’ comments**

Dear Editor,

We thank you for providing the opportunity to revise and submit the manuscript. We are thankful to the reviewers for their feedbacks and comments to improve the manuscript. We sincerely appreciate their valuable repeated efforts for providing their valid and constructive comments in order to improve manuscript. We agreed with suggestions accordingly and changes reflected with text highlighted in track changes.

Here is our pointwise response the reviewers’ comments and concerns.

| Original comments of the reviewers | Response by the authors |
| --- | --- |
| In the Introduction, from line 95 to 99, please clarify that you are describing your own previous work. This is important for transparency, and to provide the background for why you are now performing this related study to further elucidate some of the knowledge gaps that remain. | It is mentioned now. |
| In your Discussion, your newly added text is a nice explanation, but it is not clear that the preceding sentence is a description of your own previous work. Consider changing "As a part of study" to "In our previous study, [ref 17]". Can you describe the findings of the current study and how they relate to those of the previous one at all? | It is changed.  It is to mention here that current study findings do not relate to previous work it is different in focus and uniqueness (as mentioned in table below). |

Dear Editor and Reviewer:

We agree that some findings (narrative quotes) from current study was used to support BMJ paper where its focus was completely different. Current paper is a standalone and independent paper (and not a duplication) and have fundamental differences with BMJ paper for the ease; we have mentioned the differences here and also discussed in discussion section. Please refer the table below;

**Difference between BMJ paper and current paper**

| **Aspect** | **BMJ Paper** | **Current Paper** |
| --- | --- | --- |
| Geographic scope | 9 countries (broader but less depth). | 5 countries (regional focus, more depth). |
| Methodology | Mixed-methods: Largely quantitative-led with limited qualitative analysis. The qualitative components were meant to support and triangulate the quantitative findings. | Qualitative: In-depth thematic analysis with extensive illustrative quotes. |
| Focus areas | Awareness, readiness, and feasibility of citizen science; comparison between analogue and digital citizen science. | Community perceptions; lived experiences; contextual factors affecting citizen science participation in the region; sustainability of citizen science. |
| Uniqueness | By focusing on which stage of readiness the participants from each country were in, this paper gives a general overview of what to focus on if policymakers and researchers want to move people up the readiness levels. This paper also focused on whether analogue or digital citizen science was preferred. | By focusing on the lived experiences of participants, this paper provides practical recommendations and contextualized insights that are actionable for policymakers and researchers in the region to improve citizen science engagement for future pandemics. |
